# Endoscopic Retrieval of an Ingested Mobile Phone From the Stomach of a Prisoner: When Gastroenterologists Answer the Call

**DOI:** 10.7759/cureus.33053

**Published:** 2022-12-28

**Authors:** Ahmed Ali, Ali M Mahgoub, Samar Emad, Ahmed H Abdelfattah

**Affiliations:** 1 Internal Medicine, Mansoura International Hospital, Mansoura, EGY; 2 Internal Medicine Department, Gastroenterology and Hepatology Unit, Mansoura University Specialized Internal Medicine Hospital, Mansoura, EGY; 3 Internal Medicine, University of Kentucky College of Medicine, Lexington, USA

**Keywords:** intentional ingestion, mobile phone ingestion, esophagogastroduodenoscopy (egd), foriegn body retrieval, foriegn body ingestion

## Abstract

Foreign body ingestion (FBI) is a common problem among the pediatric population. The intentional ingestion of foreign bodies in the adult population is common among psychiatric patients, patients with developmental delay, alcohol use disorder, and prisoners. The management of complex FBI cases like mobile phones is not standardized in the literature. The care was discussed in a few case reports, and till the end of 2020, there were only four cases reported. We present this rare case of mobile phone ingestion, which was successfully managed by upper esophagogastroduodenoscopy (EGD) without the need for surgical intervention.

## Introduction

Foreign body ingestion (FBI) is a common emergency in the gastrointestinal (GI) practice. Most foreign body ingestions are reported in the pediatric population (around 80% of the cases). FBI in adult patients is not very common and it is usually reported in psychiatric patients and prisoners [1]. Intentional FBI among prisoners can be seen commonly for secondary gain, for example, it was reported that 1 out of 1900 inmates in the Ohio state had FBI [2]. Most of FBI cases can be managed conservatively without intervention, only 10 to 20% of the cases would need endoscopic retrieval [1]. Complex foreign bodies like mobile phones don’t have a standardized approach, therefore the importance of reporting the individual experience in such rare cases. Here we report a case of successful retrieval of a mobile phone after 40 days of intentional ingestion.

## Case presentation

**Figure 1:**
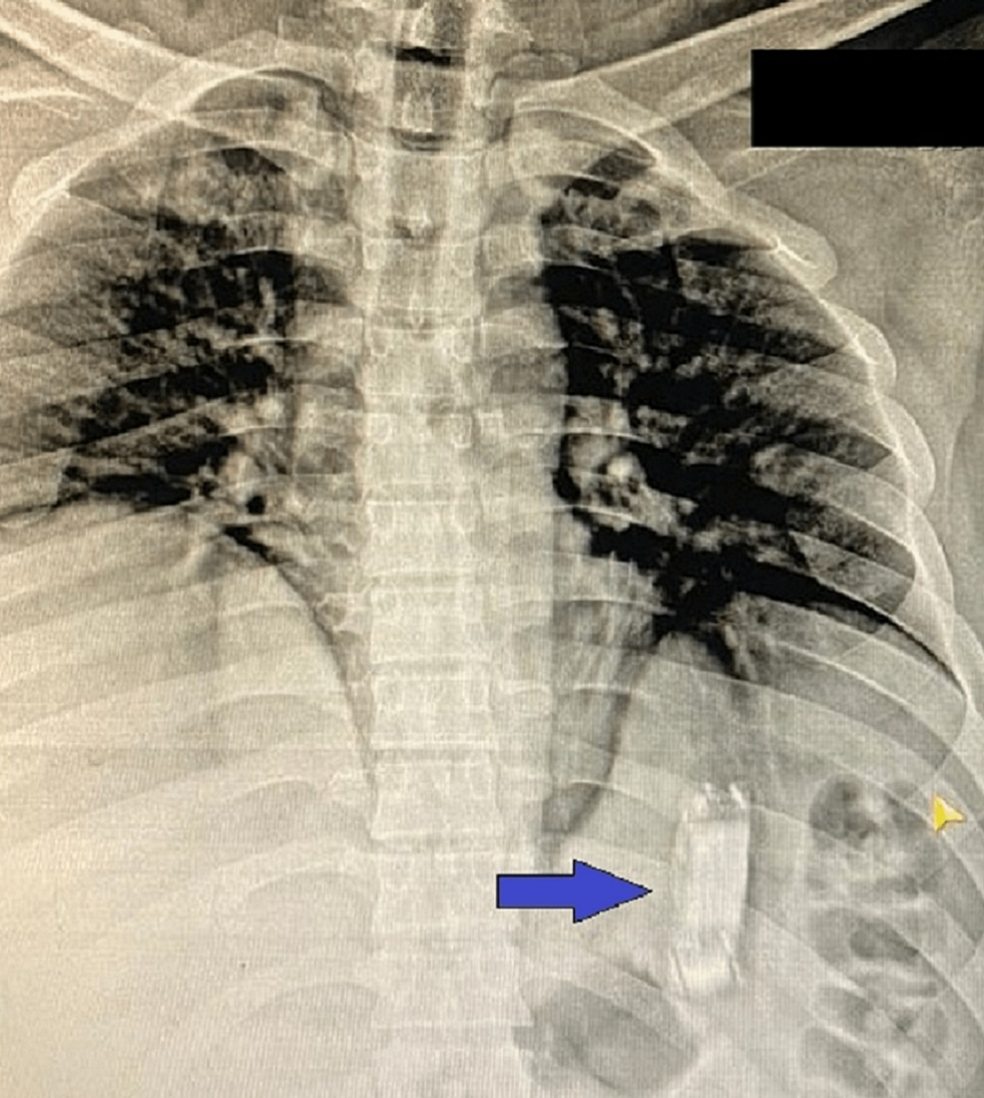
X-ray of chest and upper abdomen, with a blue arrow pointing to the mobile phone in the stomach

We present a case of a 35-year-old male prisoner who was presented to our emergency department (ED) with intentional mobile phone ingestion 40 days before. The patient did not have a significant past medical history, he did not have any chronic disease or chronic medications needed. Social history was significant for smoking and cannabis usage. On presentation, he did not have any symptoms or signs like abdominal pain, discomfort, nausea, or vomiting. Physical examination was completely normal with stable vital signs. . We did an erect chest and abdominal X-ray in the ED to confirm the site of the mobile phone. The stomach looked distended but there was no free gas under the diaphragm to suggest perforation (Figure 1).

**Figure 2:**
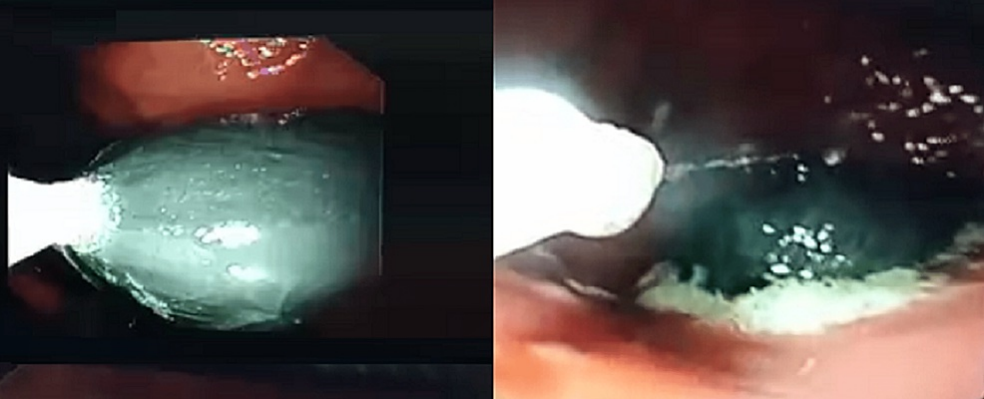
Endoscopic view of the mobile phone in a plastic packet

**Figure 3:**
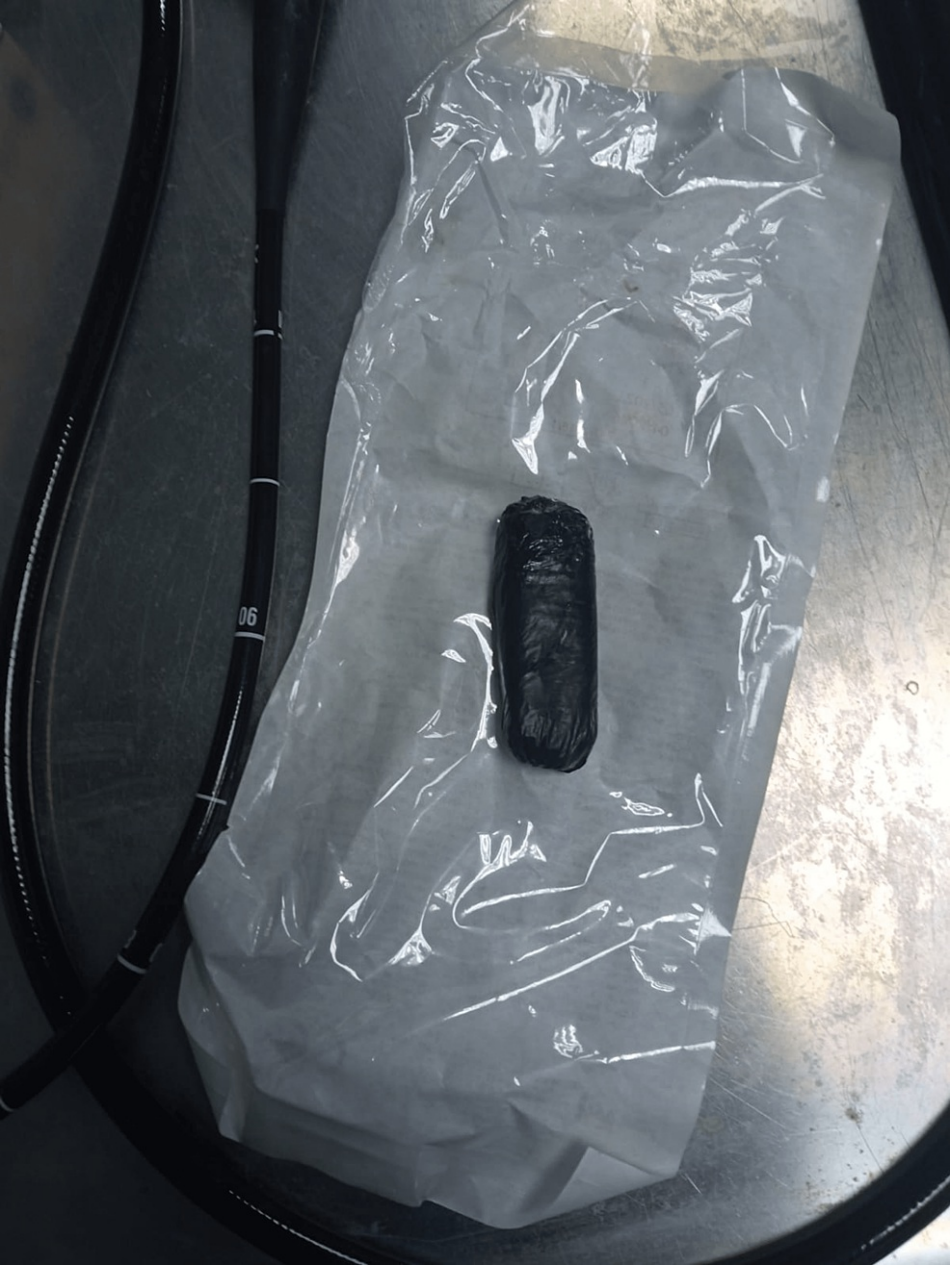
The mobile phone in a plastic packet after successful retrieval.

It measured about 5.7 cm x 2.5 cm on the X-ray. The discussion was made with the patient about the need for endoscopic retrieval and the possibility of surgical intervention in case of endoscopic approach failure. The patient gave consent for both. Upper esophagogastroduodenoscopy (EGD) was done with the help of general anesthesia. First, the patient was in the left lateral position, but the retrieval was not successful using this position. So, the patient was put on his back, and Boston 33 mm oval snare was used to grip it (Figure 2). The phone was retrieved successfully in this position by snaring the tip of the phone plastic packet (Figure 3). The patient recovered in a very good way without any post-procedure complications. Psychiatric help was offered to the patient, but the patient declined it. The patient was discharged later in the evening successfully after a short period of observation in the ED.

## Discussion

Foreign body ingestion is a common problem encountered by gastroenterologists everywhere. It can be seen in all age groups with most cases being reported among children [1]. Whether it’s a child who finds a coin interesting, so he puts it in his mouth out of curiosity and accidentally swallows it, or an elderly ingesting his own dentures by mistake, most of the time FBI is unintentional. However, intentional FBI is still seen especially in psychiatric patients and prisoners. About 90% of foreign bodies pass through the GI tract without complications and don’t necessarily require intervention to remove them [1]. The rule is that if the object passes successfully through the esophagus, it will negotiate its way easily through the rest of the GI tract without causing harm. There are some exceptions where an emergent/urgent endoscopic or even surgical intervention may be required to retrieve these objects as in the case of sharp, large, or long objects, or longstanding FBI more than 24 hours [3]. In such cases, there’s a risk of perforation, fistula formation, abscess, mediastinitis, or peritonitis, and intervention is needed [4].

Some cases of the FBI may require plain radiography assessment for the neck, chest, and abdomen to localize the foreign body, in case of a radio-opaque foreign body further follow-up assessment may be needed to detect its movement and follow up its exit from the body [4].

Gastric foreign bodies can be managed conservatively by serial physical examinations, watchful monitoring, and sometimes radiological assessments in most cases without the need for hospital admission [5]. The notable exceptions are objects longer than 5 cm or wider than 2.5 cm as such objects are unlikely to pass through the pylorus and require endoscopic retrieval [1,5]. Large foreign bodies can be complicated by impaction at the cricopharyngeal, gastro-esophageal junction, pylorus, and the ileocecal valve or bowel obstruction which requires surgical laparotomy [4].

In our case, the patient intentionally ingested a mobile phone trying to smuggle it but because it was 5.7 cm x 2.5 cm the phone didn’t pass through the pylorus and remained in the stomach for nearly six weeks. Given the patient’s circumstances being a prisoner making the arrangement for an elective endoscopy a harder task than usual, the decision was made to carry out upper GI endoscopy without delay and the mobile phone was successfully retrieved without complications. A mobile phone is a complex foreign body as it contains a battery inside it which can cause multiple complications like gastric irritation, ulceration, or perforation [6]. Our patient wrapped it in multiple plastic layers which prevented those possible complications.

We could only find very few case reports of mobile phone ingestion which were managed endoscopically during the review of available literature; some other patients needed surgical intervention [7]. To the best of our knowledge, this is the first case to be reported in Egypt.

## Conclusions

Mobile phone ingestion is a very rare encounter in clinical practice without a standardized approach. It can be managed endoscopically with an expert endoscopist, with the need for surgical intervention in some cases. It is important to know the size and dimensions of the ingested foreign body before endoscopic intervention to avoid complications like viscous rupture or perforation. 
